# Immunogenicity and effectiveness of a bivalent influenza A/H1N2 vaccine strain against seasonal human influenza A viruses in mice

**DOI:** 10.1186/s43141-022-00436-y

**Published:** 2022-11-04

**Authors:** Mina Nabil Kamel, Sara H. Mahmoud, Yassmin Moatasim, Ahmed El Taweel, Mahmoud Shehata, Mohamed Refaat Shehata, Elsayed Tarek AbdElSalam, Mohamed A. Ali, Ahmed Mostafa

**Affiliations:** 1 Center of Scientific Excellence for Influenza Viruses, National Research Centre, Giza, 12622 Egypt; 2 Chemistry Department, Faculty of Science, Cairo University, Giza, Egypt; 3 Department of Botany and Microbiology, Faculty of Science, Cairo University, Gamaa Street, Giza, 12613 Egypt

**Keywords:** Influenza, Vaccine effectiveness, Immunogenicity, Influenza A/H1N2, Influenza A/H1N1, Influenza A/H3N2

## Abstract

**Background:**

Recent studies and reports have documented the ability of the co-circulating seasonal influenza A/H1N1 (ancestor: 2009 pandemic H1N1) and A/H3N2 to exchange their genetic segments, generating a novel H1N2 strain in different geographical localities around the world with an ability to infect human. This raises concerns and triggers alarms to develop a multivalent vaccine that can protect against the documented H1- and H3-type human influenza A viruses (IAVs).

**Results:**

Here, we generated a PR8-based vaccine strain that carries the HA gene segment from the contemporary H1N1 virus while the NA gene segment was derived from a currently circulating influenza A/H3N2 strain. A recombinant PR8-based H1N2 vaccine strain (rgH1N2), engineered by reassortment between influenza A/H1N1 and A/H3N2 to mimic the documented human influenza A/H1N2, was used for immunization to provoke immunogenicity and cross-antigenicity against the H1- and H3-type human IAVs and was evaluated for its immunogenicity and effectiveness in mice. Following challenge infection of rgH1N2-vaccinated mice with contemporary influenza A/H1N1 and A/H3N2, results revealed that rgH1N2-vaccinated mice showed less viral shedding, more survival, and less body weight loss compared to control unvaccinated groups and vaccinated mice with rgH1N1 and rgH3N2.

**Conclusions:**

This study highlights the applicability of the PR8-based H1N2 vaccine strain to protect against seasonal IAVs and emphasizes the role of both surface proteins, HA and NA, to stimulate protective and neutralizing antibodies against circulating influenza A/H1N1 and A/H3N2 strains.

## Background

Influenza A virus (IAV) particles encapsulate segmented RNA genome, allowing viral segments exchange “reassortment” to generate a variety of nascent reassortant viruses [1]. Nowadays, all circulating human IAVs carry one or more gene segments from the pandemic IAVs of the last century especially the ongoing seasonal versions of the ancestor pandemic strains, H1N1 and H3N2 [1].

The co-circulation of influenza A/H1N1 and A/H3N2 strains since 1968 led frequently to the emergence of a new subtype of IAV, namely influenza A/H1N2. This reassortment event was firstly reported in China between December 1988 and March 1989 [2, 3]. Between 2000 and 2002, influenza A/H1N2 re-emerged again as sporadic H1-type-positive cases in several geographical localities like Thailand, Singapore, Malaysia, and Indonesia [4], while in the UK, influenza A/H1N2 virus was reported in humans for the first time during flu season 2001–2002 as an outbreak. Interestingly, H1N2 was the most predominant during this flu season, overcoming the influenza A/H1N1 and A/H3N2 with a positivity rate of 54% of all IAV-positive samples [5].

The first reassortment between seasonal influenza 2009 pandemic influenza A/H1N1 and seasonal influenza A/H3N2 was reported in India in late 2009 [6]. This was followed by sporadic detection of clinical influenza A/H1N2 in humans in several countries including four human H1N2 cases in the USA in 2012 [7] and a human H1N2 case in 2018 in the Netherlands [8]. Interestingly, the influenza A/H1N2 in the Netherlands showed a distinct genetic makeup, including PB2, PB1, PA, NP, NA, and M segments from influenza A/H3N2 and HA and NS segments from A(H1N1)pdm09 [8]. Till 2019, all documented reports showed that influenza A/H3N2 acts as an acceptor while influenza A/H1N1 acts as a donor. In contrast, influenza A/H1N2 genetic composition in Sweden was reported to obtain the neuraminidase gene from influenza A/H3N2 and other genes from influenza A/H1N1 [9]. In late 2020, an influenza A/H1N2-infected case was reported in Brazil [10]. Nowadays, influenza A/H1N2 is mainly genetically constructed from influenza A/H1N1 and contracted only the neuraminidase from influenza A/H3N2. This raises the concern of an increased probability of circulation within the human population [3].

Vaccination is still the primary defense line to combat IAV infection [11]. Reverse genetics (rg) systems have been employed to rescue recombinant influenza vaccine strains (IVS) in a timely and controlled manner during seasonal epidemics or pandemics to overcome [1, 12–15]. This frequent emergence of reassortant influenza A/H1N2 alarms for preparedness to control it in case it emerges as a pandemic strain. Moreover, rare studies have tested the applicability of heterogenous influenza A/H1N2 as a multivalent vaccine against all human H1- and H3-type IAVs.

In this study, we aim to use a generated PR8-based influenza A/H1N2 virus as a candidate vaccine strain to provide multiple protection against influenza A/H1N1, A/H3N2, and A/H1N2 viruses in vaccinated mice.

## Methods

### Cells and viruses

The Madin–Darby canine kidney (MDCK) cells and the 293T human embryonic kidney cells expressing the SV40 large T-antigen were maintained in Dulbecco’s modified Eagle medium (DMEM; Invitrogen), supplemented with 10% fetal bovine serum (FBS, Invitrogen), penicillin (Pen, 100 IU/ml), and streptomycin (Strep, 100 μg/ml) (Pen/Strep). All cells were cultured and stored in a humidified incubator at 37°C and 5% CO_2_. Human influenza A/H1N1 (A/Egypt/NRC098/2019 (H1N1)), A/H3N2 (A/Egypt/NRC107/2019 (H3N2)), and A/Puerto Rico/8/1934 (H1N1, PR8) viruses were obtained from the virus stocks of the Center of Scientific Excellence for Influenza Viruses, National Research Centre, Egypt. To prepare virus stocks, both viruses were propagated in MDCK cells as previously described [16].

### Phylogenetic analysis

The evolutionary history was inferred using the neighbor-joining method [17]. The percentage of replicate trees in which the associated taxa clustered together in the bootstrap test (1000 replicates) are shown next to the branches [18]. The evolutionary distances were computed using the maximum composite likelihood method [19]. Phylogenetic analyses were conducted using MEGA-6 software [20].

### Plasmids and reverse genetics

The HA and NA gene segments from influenza human influenza A/H1N1 (A/Egypt/NRC098/2019 (H1N1)) and A/H3N2 (A/Egypt/NRC107/2019 (H3N2)) viruses were amplified by reverse transcription polymerase chain reaction (RT-PCR) using universal primers for reverse genetics (rg) of IAV [21]. The purified PCR product of each viral segment was digested with either BsmBI for the HA segment or BsaI for the NA segment and then individually ligated with an AarI-linearized pMP*ccd*B vector as previously described [16]. The correct constructs, as confirmed by enzymatic digestion and sequencing, were subsequently used to generate recombinant rg viruses in the genetic background of well-replicating influenza A/Puerto Rico/8/1934 (H1N1, PR8).

**Fig. 1:**
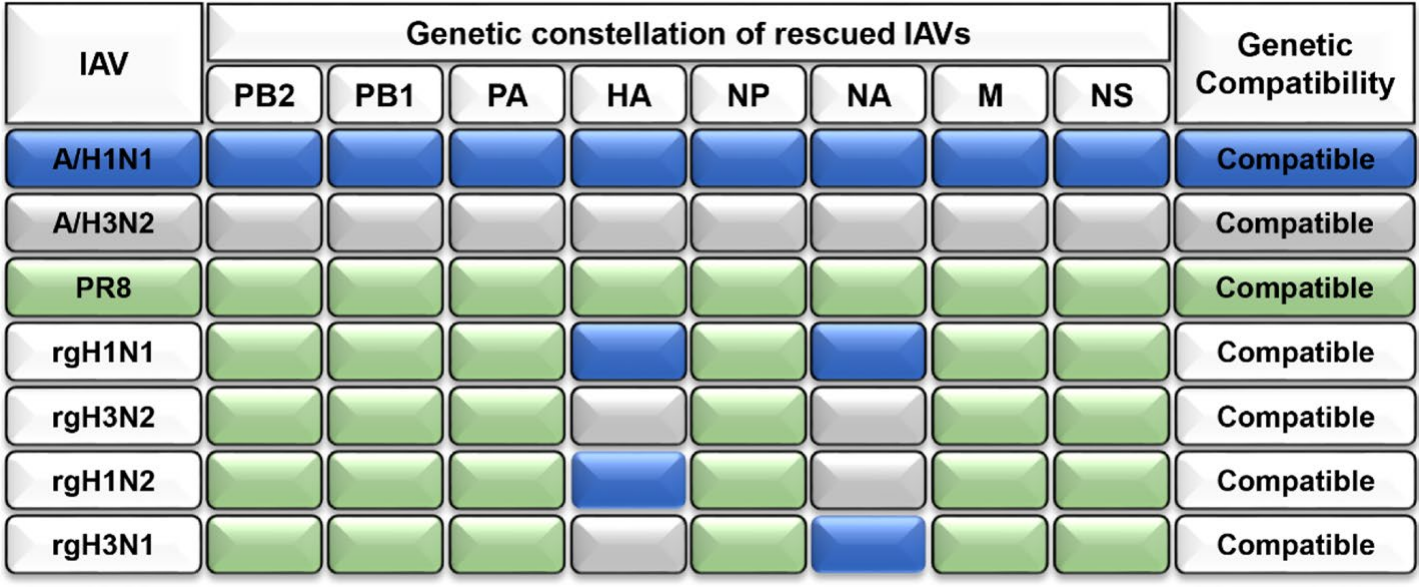
Genetic illustration of rescued viruses that were tested as candidate vaccine seed strains and their parent strains

Rescue of 6+2 rg viruses which are genetically composed of 6 internal genes from A/Puerto Rico/8/1934 (H1N1, PR8) and surface glycoproteins (HA and NA) from seasonal influenza A/H1N1 was done to generate the rgH1N1 vaccine strain and their counterparts from seasonal influenza A/H3N2 to generate the rgH3N2 vaccine strain and then the surface glycoprotein from the seasonal IAVs was shuffled to generate rgH1N2 and rgH3N1 candidate vaccine strains. The genetic constellations of the parental and rescued viruses are listed in Fig. 1.

### Virus propagation, titration, and antigen preparation

Virus stock was propagated in MDCK cells cultured in a T-175 flask (Greiner bio-one GmbH, Germany); then, cells were microscopically investigated daily. The virus-infected culture supernatant was clarified by centrifugation at 2500 rpm for 15 min at 4°C twice. The harvested virus was titrated by plaque titration assay. Harvested influenza A/H1N1, A/H3N2, rgH1N1, rgH3N2, rgH1N2, and rgH3N1 were inactivated with 0.1% β-propiolactone (BPL) (Sigma-Aldrich); then, the treated virus was incubated at 4°C for 2 days in a cooling shaking incubator. The β-propiolactone-treated viruses were tested for loss of viral infectivity by inoculating them into MDCK monolayers for up to 5 days. No cytopathic effect (CPE) was observed on MDCK cells that were inoculated with inactivated viruses. The inactivated viruses were further concentrated by ultracentrifugation (80,000×g, 4 °C, 1 h with 20% sucrose as a cushion). Total protein content was measured by the Pierce™ BCA Protein Assay Kit (Thermo Fisher Scientific).

### Preparation of inactivated candidate vaccine

The desired concentration (15 μg) was diluted in 1× PBS and then mixed with Imject Alum adjuvant (Invitrogen) in a ratio of 2:1 (v/v). The formulated inactivated vaccines plus adjuvant were mixed for 30 min under cooling conditions to confirm the adsorption of viral antigen into the surface of the alum.

### Immunogenicity of the generated H1- and H3-type vaccine strains (VS)

**Fig. 2:**
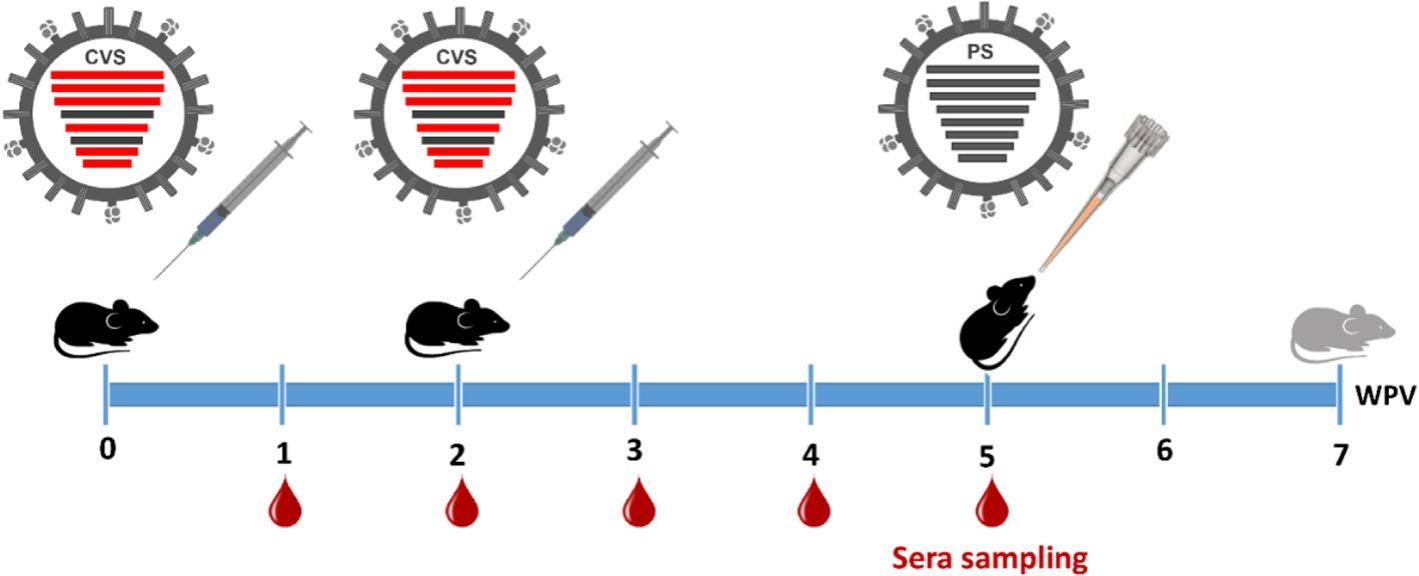
The experimental timeline includes initial immunostimulation, booster vaccination, and challenge infection of immunized mice for up to 7 weeks post-vaccination (WPV). Eight-week-old black/6 mice were immunized at zero time of the experiment, followed by a booster vaccination at week 2 post-initial immunization to stimulate neutralizing antibodies against candidate vaccine strain (CVS) in mice. Challenge infection of immunized mice with the parent strains (PS) was done at week 5 post-vaccination and followed up for 2 weeks post-infection

To evaluate the immunogenicity of the generated vaccine strains, 8-week-old black/6 mice were used. Mice were obtained from the Animal House Colony of the National Research Centre (NRC), Egypt. All mice were verified to be seronegative for H1 and H3 antibodies using the plaque reduction neutralization assay (PRNT). The animals were maintained at a controlled temperature of 24 ± 1 °C with a 12–12-h light-dark cycle (light cycle, 07:00–19:00) and were allowed free access to water and standard feed ad libitum. Animals were allocated into six groups (*n* = 16) (rgH1N2, rgH3N1, rgH1N1, rgH3N2, control non-infected, and control infected). As illustrated in Fig. 2, black/6 mice were intramuscularly (IM) immunized with 50 μl of inactivated vaccine strain (VS) containing 15 μg viral antigen(s) of each inactivated virus. In the second-week post-vaccination, a subsequent booster dose was administrated with the same composition for each group. The animals were then followed up for 3 weeks post-vaccination (WPV). All animal sera were separated and stored at −20 °C until used. Serum samples were collected weekly from immunized animals until week 5 post-vaccination.

### Plaque reduction neutralization assay (PRNT)

MDCK cells were seeded in 6-well plates at a cell count of 1 × 10^5^ cells/well and incubated overnight at 37°C and 5% CO_2_. Subsequently, collected sera were diluted to 1:10, followed by bifold serial dilutions using DMEM media containing 0.2% bovine serum albumin (BSA) and 1% Pen/Strep mixture, mixed with either influenza A/H1N1 or A/H3N2, and incubated for 1h to allow neutralization. Following cell-free neutralization, the mixtures of diluted sera/virus were transferred into MDCK cell monolayers to all viral adsorption of non-neutralized viral particles and incubated at RT for 1 h. The inocula containing mixture residuals were aspirated, and cells were overlaid with an overlay composite containing 1% agarose, 1× MEM, 0.2% bovine serum albumin (BSA), 1% Pen/Strep mixture, and TPCK-trypsin (1 μg/ml) for 72 h. Cell monolayers were then fixed with 10% paraformaldehyde, and plaques were visualized using 1% crystal violet solution and counted to calculate viral reduction (%). PRNT endpoint titers were expressed as the reciprocal of the last serum dilution. The PRNT titer was calculated based on a 50% reduction in plaque count (PRNT_50_).

### Challenge infection

At week 5 post-vaccination, each vaccinated group was separated into 2 subgroups (8 mice/each) for nasal challenge infection by the parental seasonal influenza A/H1N1 or A/H3N2 viruses (Fig. 2). The virus concentration in the inoculum was unified at 5log_10_ pfu/20 μl. Mice were inoculated by 20 μl intranasally. Mice were monitored daily within 14 days post-infection (dpi) for mortality and morbidity. Nasal wash samples were obtained from each mouse, using the saline solution on days 3, 5, and 7 post-infection for virus titration using quantitative real-time RT-PCR of the influenza M gene [10]. On day 3 post-infection, 3 mice from each subgroup were dissected, and the lung and nasal termination were collected. Organ homogenization was done by using a TissueLyser at 1/1 weight/volume for 30 s at 50 Hz/s. The supernatant was collected and titration was performed by using quantitative real-time RT-PCR as described previously [10].

### Ethical statement and biosafety

All animal experiments and procedures were conducted following the guidelines and regulations approved by the Medical Research Ethics Committee (MREC) of the National Research Centre (NRC), Egypt (permission code: 19–274). All experiments involving IAVs were performed using biosafety level 2 laboratories and isolators approved for such use by the local authorities (NRC, Giza, Egypt).

### Statistical data analysis

Statistical data analysis was done by using GraphPad Prism V5 (GraphPad Inc., San Diego, CA, USA). A one-way ANOVA test was performed, followed by Tukey’s post hoc test. Data were represented as mean ± SD. *P* values of ≤0.05 were considered statistically significant.

## Results

### Characterization of isolated influenza A/H1N1 and A/H3N2 viruses used to generate studied candidate vaccine strains against H1- and H3-type human IAVs

Candidate vaccine viruses (CVVs) are usually selected by the end of the influenza season to protect against the viruses that are likely to circulate during the upcoming influenza season. Herein, two fully characterized viral human influenza A isolates, A/Egypt/NRC098/2019 (H1N1) (GSAID number: EPI_ISL_12995118) and A/Egypt/NRC107/2019 (H3N2) (GSAID number: EPI_ISL_12995401), representing the circulating seasonal IAVs were selected to generate a panel of PR8-based candidate vaccine strains.

**Fig. 3:**
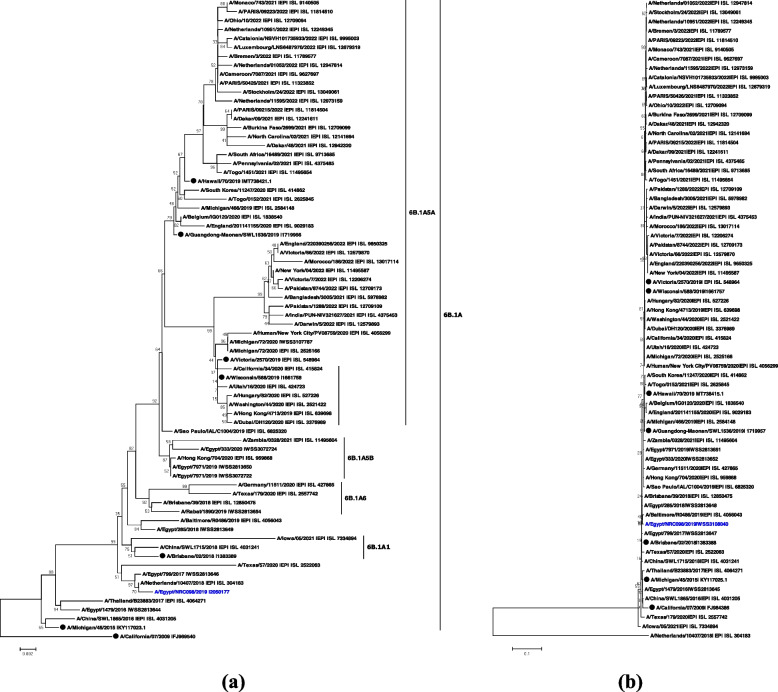
Phylogenetic analysis of the full HA (**a**) and NA (**b**) genetic segments from the studied candidate influenza vaccine strain A/Egypt/NRC098/2019 (H1N1). The tree was rooted with A/California/07/2009. Our candidate vaccine strain A/Egypt/NRC098/2019 (H1N1) is indicated in blue color. Vaccine strains A/Brisbane/02/2018 (egg-based, 2019–2020 NH influenza season), A/Guangdong-Maonan/SWL1536/2019 (egg-based, 2020–2021 influenza season), A/Hawaii/70/2019 (cell-based, 2020–2021 influenza season), A/Victoria/2570/2019 (egg-based, 2021–2022 influenza season), and A/Wisconsin/588/2019 (cell-based, 2021–2022 influenza season) are indicated with black circles

**Fig. 4:**
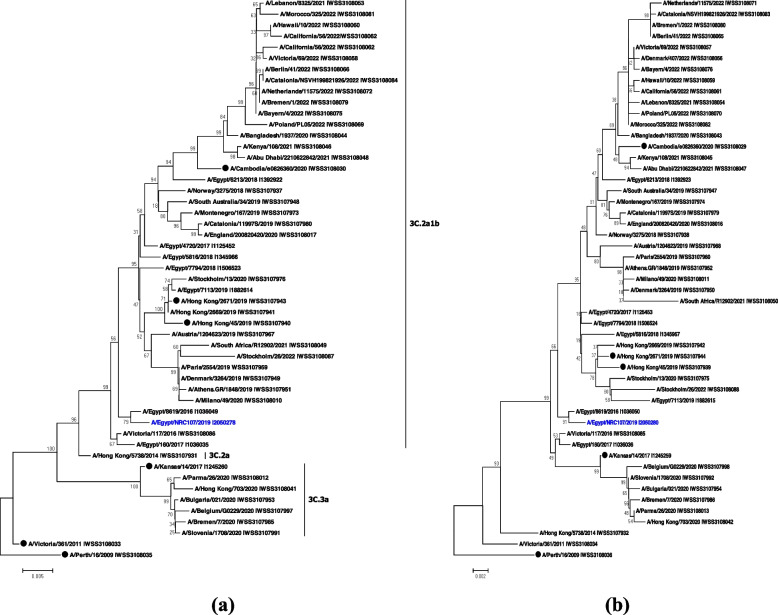
Phylogenetic analysis of the full HA (**a**) and NA (**b**) sequences from the studied candidate influenza vaccine strain A/Egypt/NRC107/2019 (H3N2). The tree is rooted with A/Perth/16/2009 (H3N2). Our studied candidate vaccine strain is indicated in blue color. Vaccine strains A/Kansas/14/2017 (egg-based, 2019–2020 season), A/Hong Kong/2671/2019 (egg-based, 2020–2021), A/Hong Kong/45/2019 (cell-based, 2020–2021 season), and A/Cambodia/e0826360/2020 (2021–2022 season) are indicated with black circles

Phylogenetic analysis of the HA and NA in comparison with the ancestor strains for influenza A/H1N1 and influenza A/H3N2, namely A/California/07/2009 (H1N1) (Fig. 3a, b) and A/Perth/16/2009 (H3N2) (Fig. 4a, b), and recently used/recommended vaccine strains by WHO, revealed the phylogenetic relation to our isolates.

### Rescue and propagation of candidate vaccine

**Fig. 5:**
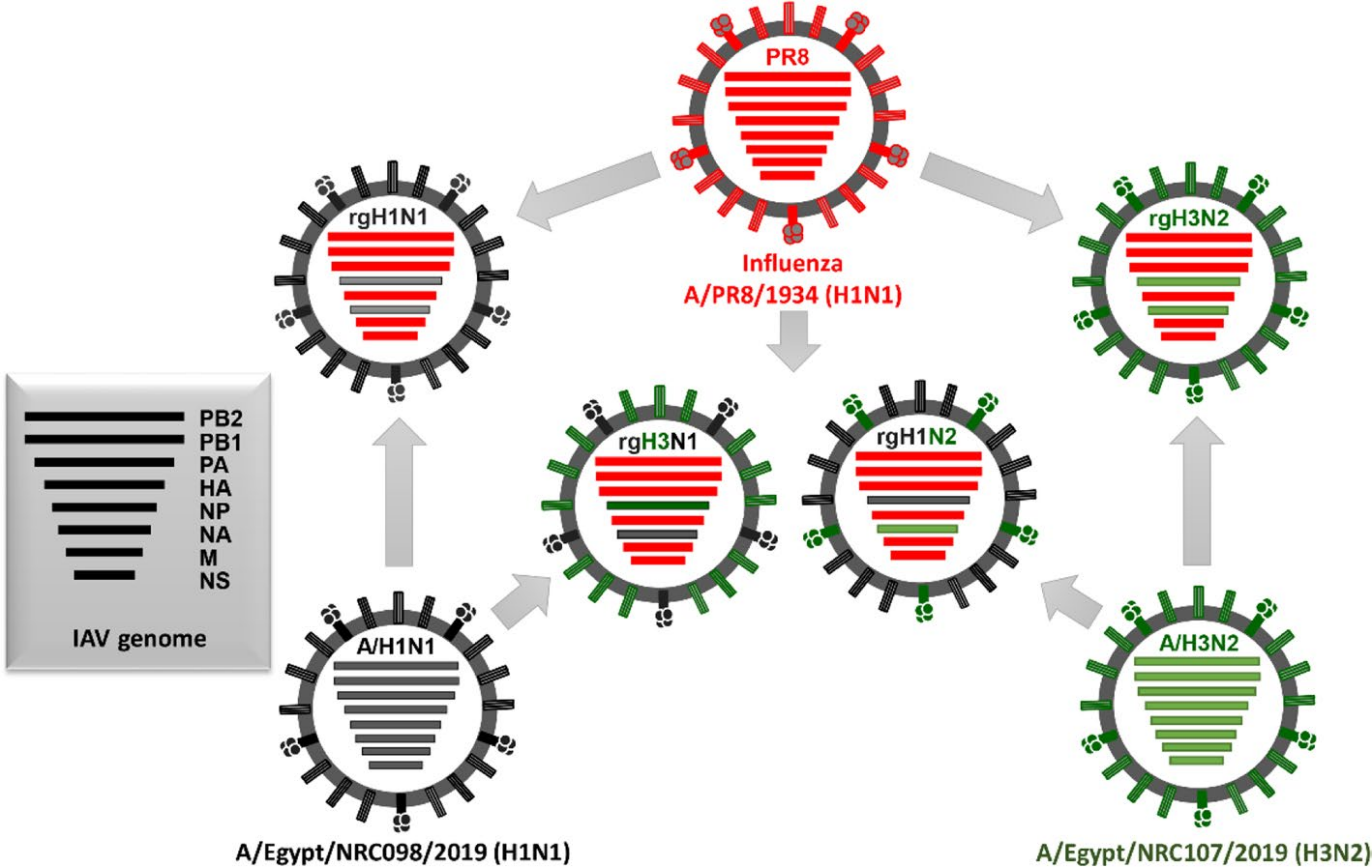
Graphical representation of the parental and rescued viruses that were tested as candidate vaccine seed strains

To evaluate the protection efficiency of the rgH1N2 and rgH3N1 compared to the classical control rgH1N1 and rgH3N2 vaccine strains, the four PR8-based vaccine strains were successfully rescued using reverse genetics (Fig. 5). The rgH1N2 acquires the internal backbone segments from PR8, the surface glycoproteins from circulating IAVs (HA from an influenza A/H1N1 and NA from an influenza A/H3N2 strain). However, the rgH3N1 acquires the HA gene segment from influenza A/H3N2 and NA from influenza A/H1N1 strains, with the other internal proteins encoding segments from PR8.

For the control PR8-based vaccine strains, rgH1N1 and rgH3N2, the two main surface glycoproteins (HA and NA) were derived from their corresponding parental strains. The genetic composition of each rescued vaccine strain was confirmed by subtyping using RT-PCR.

Historically, candidate vaccine viruses are required to be isolated and grown in chicken eggs or certain mammalian cell cultures including Madin–Darby canine kidney (MDCK) cells or African green monkey kidney (Vero) cells. Expectedly, the influenza A/Egypt/NRC107/2019 (H3N2) virus grows poorly in eggs, making it challenging to get a good candidate vaccine strain for vaccine production. It grows well in MDCK cells (virus titer = 2.3 × 10^7^ PFU/ml). Accordingly, the rgH1N1, rgH3N2, rgH1N2, and rgH3N1 were propagated in mammalian MDCK cell cultures. Nevertheless, the rgH1N1, rgH1N2, and rgH3N1 showed good titers when propagated in embryonated eggs at 36 h post-inoculation (data not shown).

### Immunogenicity of reassortant candidate vaccine strains

**Fig. 6:**
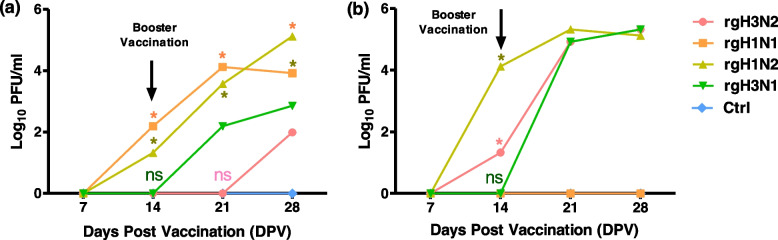
Immunogenicity of inactivated candidate vaccine strains as measured by plaque reduction neutralization assay (PRNT) for the collected sera from vaccinated and control mice groups (*n* = 16 per group) at different time points post-vaccination. The sera plaque neutralization assay was done against influenza A/H1N1 (**a**) and A/H3N2 (**b**). Statistical analysis was performed using one-way ANOVA followed by Tukey’s post hoc test. The significant differences are indicated (*P < 0.05 and non-significant = ns)

Black/6 mice were immunized by candidate vaccine strains (rgH1N1, rgH3N2, rgH3N1, or rgH1N2) via the intramuscular route (15 μg/dose), followed by administration of the same dose 2 weeks post-vaccination as a booster vaccination. Collected serum samples were analyzed weekly as illustrated in Fig. 2 every week to monitor the immunogenicity in immunized animals till the 4th-week post-vaccination (WPV) (Fig. 6).

Plaque neutralization assay for sera collected from immunized mice showed that the groups vaccinated by either rgH1N1 or rgH1N2 showed antibody titer within the second-week post-vaccination while the group vaccinated by rgH3N1 showed antibody titer within the third-week post-vaccination against influenza A/H1N1. In contrast, the groups which were vaccinated by either rgH3N2 or rgH3N1 or rgH1N2 showed antibody titer within the second-week post-vaccination against influenza A/H3N2. This confirms the high immunogenicity of the reassortant rgH1N2 vaccine strain and its ability to stimulate neutralizing antibodies against both parental strains, with comparable higher efficiency than the control vaccine strains.

### Vaccine effectiveness following challenge infection

To confirm that the stimulated neutralizing antibodies can neutralize the virus and disrupt the viral infection with circulating strains, challenge infection of the vaccinated mice occurred using circulating seasonal IAVs. After 4 weeks post-vaccination, each group was divided into two subgroups for challenge infection. One subgroup was challenged with the parental influenza A/H1N1 virus, while the other subgroup was challenged with the parental influenza A/H3N2 virus. The virus concentration that was used for the challenge infection was set at 5log_10_ PFU/20 μl. This dose was consistent with the viral infectious dose used in previous pathogenicity studies of influenza A/H3N2 [22] and A/H1N1 [23].

**Fig. 7:**
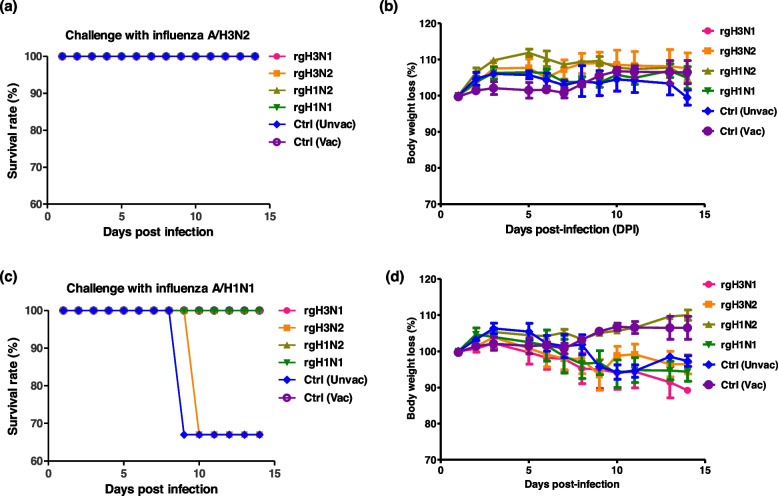
Survival rate and body weight losses of vaccinated mice (*n* = 8 per each virus challenge) against circulating IAVs. Vaccinated mice that were infected with influenza A/H3N2 followed by monitoring the survival rate percent (**a**) and body weight loss percent (**b**). The second group of vaccinated mice was challenged with influenza A/H1N1 and protectiveness was investigated by following up on the survival rate percent (**c**) and body weight loss percent (**d**)

All groups of mice challenged with influenza A/H3N2 survived with a non-significant difference in body weight loss (Fig. 7a, b), while mice challenged by influenza A/H1N1 showed that unvaccinated challenged mice and those vaccinated with rgH3N2 have a survival rate of 67% without a significant difference in body weight loss (Fig. 7c, d). Interestingly, rgH1N2-vaccinated mice and control (uninfected) groups showed a comparable pattern in body weight gain, suggesting that those rgH1N2-vaccinated mice are well protected against infection with seasonal influenza A/H1N1 challenge strain.

### Impact of vaccination with candidate vaccine strains on viral shedding

**Fig. 8:**
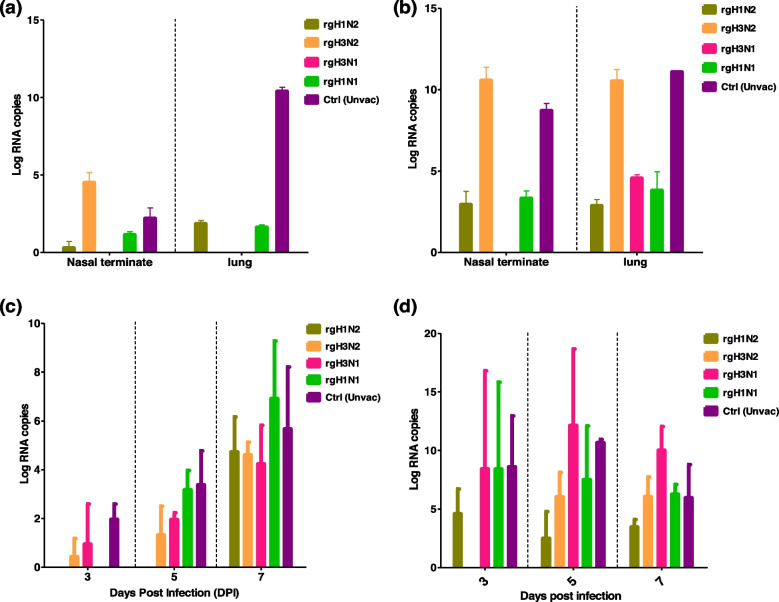
Viral shedding or viral loads in lungs, nasal terminates, and nasal washes of the vaccinated and control mice (*n* = 8). The viral loads in the lungs and nasal terminates were detected in challenged mice with influenza A/H3N2 (**a**) and A/H1N1 (**b**) at day 3 post-infection. The viral loads in nasal washes were detected on days 3, 5, and 7 post-infection with H3N2 (**c**) or H1N1 (**d**) using semi-quantitative rtRT-PCR

Viral loads in the lungs and nasal termination of the dissected immunized mice post-challenge infection were determined following homogenization using quantitative rtRT-PCR (Fig. 8). The lowest viral shedding in both lung and nasal turbinates in mouse groups that were challenged by influenza A/H3N2 was detected in nasal terminates and lungs of rgH1N2-vaccinated mice (Fig. 8a). In line with these results, the mouse groups immunized with rgH3N1, rgH1N2, or rgH1N1 and challenged with influenza A/H1N1 showed the lowest viral shedding among all influenza A/H1N1-infected mice. Compared to other vaccinated groups, the rgH3N2-vaccinated mice showed higher viral loads that were comparable to non-vaccinated control when challenged with influenza A/H1N1 strain (Fig. 8b).

To estimate the viral loads in the nasal washes of the challenged vaccinated and control groups, nasal washing was applied for all groups on days 3, 5, and 7 post-challenge and titrated for the viral loads by quantitative rtRT-PCR. The rgH1N2-vaccinated mice showed lower viral shedding post-infection, compared to the rgH1N1- and rgH3N2-vaccinated and control non-vaccinated mice (Fig. 8c, d).

## Discussion

Influenza A viruses (IAVs) are known for their high mutation rates due to the non-proof-reading polymerase activity of the influenza polymerase complex. Besides, the segmented nature of the IAV genome leads to genetic segment exchange or reassortment among replicating viruses in the host cell. Two main human IAVs are circulating among human populations for decades, namely influenza A/H1N1 and A/H3N2. The co-circulation of both subtypes leads occasionally to the emergence of a new human subtype, namely influenza A/H1N2, in different geographical localities around the world. Currently, two surface glycoproteins, HA and NA, are used in vaccine production because they are hosting the main antigenic sites required to induce a robust humoral immune response. Besides, studies showed that the NA can stimulate the humoral and cellular immune responses [24]. Until now, the standard WHO-recommended vaccines against seasonal IAVs are based on three or four human influenza viruses including two human IAVs.

The occasional occurrence of influenza A/H1N2 and the challenges of the current seasonal influenza vaccine strains, including difficulties in production within reasonable times before flu season, urged us to study the applicability of a hybrid vaccine strain from the two seasonal influenza viruses as a bivalent vaccine strain. To this point, we selected two seasonal influenza viruses as a representative for currently circulating lineages of influenza A/H1N1 and A/H3N2 to generate a panel of classical and hybrid PR8-based vaccine strains. Consistent with our rationale, the incidence of influenza A/H1N2 as a reassortant subtype of both seasonal influenza viruses might potentiate the necessity of generating a multivalent vaccine strain to control the three human influenza subtypes.

Several studies suggest that antibodies against the influenza surface neuraminidase (NA) protein led to protection against infection with the corresponding IAVs. Due to the low immune selective pressure on NA and the low mutation rate compared to HA, the applicability of the NA-based vaccine as a protective agent against IAV infection was highlighted [25].

On the other hand, pigs and humans share the same pattern of influenza receptors in their respiratory tract, and the inter-species transmission of influenza A viruses from pigs to humans and humans to pigs occurs in both directions [1]. Therefore, the development and use of this A/H1N2 vaccine strain in the veterinary field could be deepened as prevention and control of swine IAVs and this may be a benefit not only for pig health, but also for human health.

## Conclusions

In this study, we developed a hybrid vaccine strain, rgH1N2, expressing the HA protein of the influenza A/H1N1 and NA protein from influenza A/H3N2, which are circulating as seasonal influenza viruses. In black/6 mice (total protein content = 15 μg), the rgH1N2 vaccine was superior to other generated hybrid and control vaccine strains to protect against viral infection with influenza A/H1N1 or A/H3N2. Despite that, both seasonal influenza viruses, especially influenza A/H3N2, showed attenuated behavior in black/6 mice, and the robust effectiveness of inactivated rgH1N2 as a bivalent vaccine was documented by the high neutralizing capacity and the low viral loads/shedding following challenge infection. This study highlights the applicability of hybrid vaccine strain rgH1N2 against seasonal IAVs.

## Data Availability

All data generated or analyzed during this study are included in this published article.

## Abbreviations

VS: Vaccine strain
CVVs: Candidate vaccine viruses
IAV: Influenza A virus
rg: Reverse genetics
PR8: Influenza A/Puerto Rico/8/1934 (H1N1)
PS: Parent strain
WPV: Weeks post-vaccination
CVS: Candidate vaccine strain
